# Perch use by laying hens in a commercial aviary^1^

**DOI:** 10.3382/ps/pew111

**Published:** 2016-03-18

**Authors:** D. L. M. Campbell, M. M. Makagon, J. C. Swanson, J. M. Siegford

**Affiliations:** Animal Behavior and Welfare Group, Department of Animal Science, Michigan State University, East Lansing, MI

**Keywords:** behavior, welfare, aviary, laying hen, perch

## Abstract

Non-cage housing systems, such as the aviary, are being implemented by the laying hen industry, including in North America, in an attempt to improve the welfare of hens. Perches are a resource that is consistently included in aviaries. Hens are strongly motivated to perch, and perching can improve leg bone strength. However, hens may prefer elevated perches, particularly at night, and thus simply providing perches is not enough to improve welfare; they must be provided in a way that allows all hens to access them. Observations of laying hens using perches and ledges (flat, solid metal shelves to assist hens’ movement between tiers) in a commercial aviary revealed variation in where hens roosted within the tiered aviary enclosure across the flock cycle (peak, mid and end of lay; *P* < 0.001 for all age points). Hens most often preferred roosting in the highest enclosure levels, leading to crowding on upper perches and ledges while perch space remained available on lower levels. Restricted access to preferable perches may cause frustration in hens, leading to welfare issues. Hens roosted more on perches at peak lay than mid and end lay (*P* < 0.001) but roosted less on ledges at peak lay than mid and end lay (*P* < 0.001). Additionally, more hens roosted on both perches and ledges in the ‘dark’ observation period compared with the number of hens roosting during the ‘light’ observation period (*P* < 0.001). Further research should look at all structural elements within the system that are used by hens for roosting, such as edges of tiers and upper wire floors, to evaluate how changes in perching preferences across the lay cycle may correlate with system design and bird-based parameters.

## INTRODUCTION

Alternative housing systems, such as the aviary and enriched colony cages, are being implemented by the laying hen industry in an attempt to improve the welfare of hens. These alternative systems provide additional space and resources for hens compared to conventional cages. Perches are a key resource that is consistently incorporated into these new housing systems; however, simply providing perches is not enough to improve welfare. Perches should be provided in a way that allows all hens equal access to suitable perches, particularly at night when hens are most motivated to perch (European Food Safety Authority Animal Health and Welfare (EFSA AHAW) Panel, 2015). Research in current commercial systems will provide valuable information as national laying hen standards are being updated in several countries (e.g., Canadian National Farm Animal Care Council Poultry Code of Practice and Australian Poultry Standards and Guidelines are currently undergoing revision).

Free-living hens roost with flockmates on tree branches at night (McBride et al., 1969; Wood-Gush et al., 1978; Blokhuis, 1984), and perching under natural conditions is considered an adaptive behavior that likely reduces predation (Wood-Gush and Duncan, 1976; Wood-Gush et al., 1978). Experimental studies have shown that hens are strongly motivated to perch at night (Olsson and Keeling, 2002), and perching that involves grasping with the foot can improve leg bone strength (Duncan et al., 1992; Barnett et al., 1997). Previous work examining the behavior of hens in housing systems with perches has shown that hens will roost on perches at night as well as use them during the day, though there is strain-to-strain variation (Faure and Jones, 1982a,b; Braastad, 1990; Duncan et al., 1992; Appleby et al., 1992, 1993; Appleby, 1995; Abrahamsson, 1996; Lambe and Scott, 1998; Newberry et al., 2001; Óden et al., 2002).

Further, hens prefer higher perches for roosting at night (Olsson and Keeling, 2000; Schrader and Müller, 2009; Brendler et al., 2014), and have been reported to only use lower perches after upper tiers are filled (Odén et al. 2002). Perching by hens housed in a group is often synchronous (Appleby, 2004) and, thus, perch space guidelines are frequently based on hen body width to ensure accommodation of all hens in a system, with 15 cm/hen being the common recommendation. However, there is also evidence that the percentage of hens in the group that will perch simultaneously increases to 99 to 100% when given 22.5 cm/hen versus 71 to 78% of hens perching when they are provided with 15 cm/bird (Duncan et al., 1992). Further, the fewest hens were recorded perching when provided only 15 cm/bird compared with treatments providing to up to 26 cm/bird (Cook et al., 2011). Thus how hens prefer to use available perch space becomes important if systems are to be developed or improved to accommodate natural perching behavior. Recent guidelines (e.g., United Egg Producers, 2010; EFSA AHAW Panel, 2015) consider bird width but also include recommendations for perch shape and material characteristics, perch positioning to facilitate movement to and from the perch, and perch height requirements.

Together, the evidence from previous studies of perching behavior suggests that simply providing perches based only the linear dimension of perch space needed to accommodate a hen's body width may not improve hen welfare for all hens as intended. As several guidelines and recommendations suggest, perch height and other characteristics must also be considered (United Egg Producers 2010; EFSA AHAW Panel, 2015). However, we do not yet know exactly what is ideal with respect to the combination of these features to enable simultaneous perching of all hens on preferred perches. Hens without access to preferred perches may experience increased frustration and thus reduced welfare (Olsson and Keeling, 2000), or perch crowding may possibly result in more falls and/or collisions, leading to bone fractures or bruising. Alternatively, if perch space at preferred heights is not sufficient to accommodate all hens, the birds may choose to roost on other elements of the system, such as solid metal ledges or on top of internal feeders, rather than moving to perches at lower levels. This is true particularly if the ledges fit the perching criteria of being elevated, provide a vantage point for surveying surroundings, and can be grasped by hens with their toes (EFSA AHAW Panel, 2015). The objective of this study was to examine the day time and night time use of perches, including use of 2 ledges provided to assist hens’ transitions between levels, across the flock cycle by laying hens housed in a commercial aviary system equipped with 3-tiered enclosures. These data will be useful for evaluating the suitability of this and other aviary designs in meeting hens perching needs.

## METHODS

### Housing

**Figure 1.**
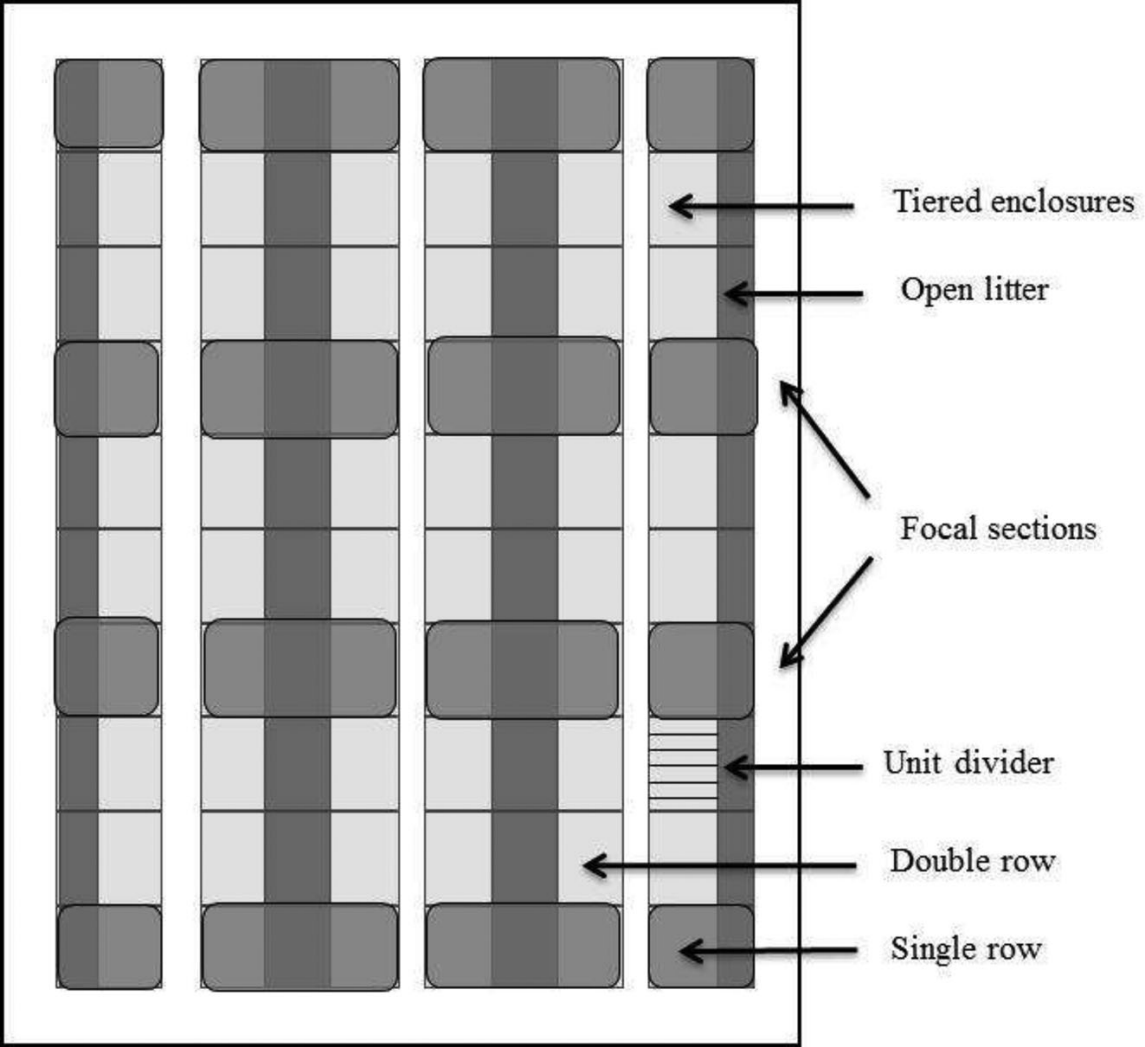
A schematic diagram of the aviary house showing the tiered enclosure and open litter area, shaded focal observational sections, unit dividers, and single and double rows.

The newly-built commercial aviary system (aviary) used in this research housed 49,677 Lohmann White laying hens. Hens were placed at 17 weeks of age (June 2012) and depopulated at 78 weeks of age (August 2013). The aviary house included 6 rows of 3-tiered enclosures, arranged as 2 outer single rows facing building walls and 2 inner double rows facing each other (Figure 1). Each row was divided along its length into 10 separate sections by wire gates (Figure 1). Tiered enclosures within each section were internally divided along their lengths into units (Figure 1), with 6 units per section in single rows and 12 units per section in double rows. (However, sections at the ends of each row had 5.5 units per section at the front end of the house and 5 units per section at the back end of the house.) Each full unit was populated with 142 hens at the start of production, for a total of 852 hens in each 6-unit single row section and a total of 1,704 hens in each 12-unit double row section. Hens could not move between unit enclosures within a section (10 fully separated sections per row of birds) when enclosure doors were closed. However, following door opening and litter access, hens could re-enter any of the units within their section, thus self-distributing into varying densities within each unit. (For additional details on available space per hen and provision of other resources, see Jones et al., 2014; Zhao et al., 2015).

**Figure 2.**
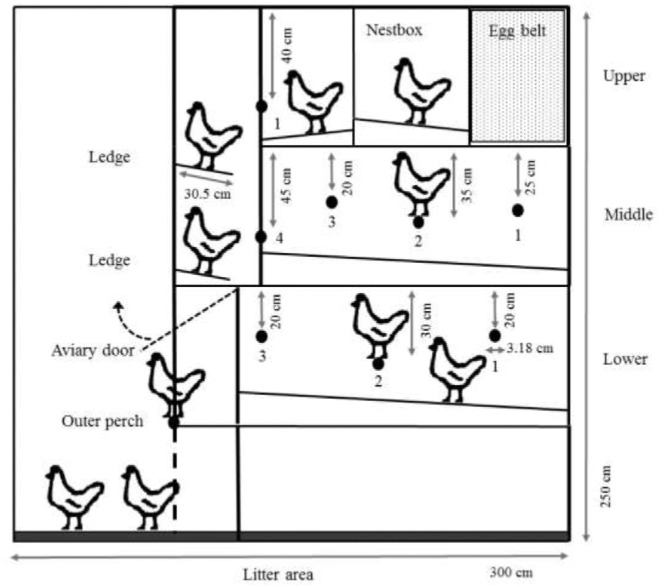
Representation of the tiered aviary enclosures indicating the numbered inner perches and ledges on each tier observed during live observations.

Each tiered aviary enclosure contained perches at all levels, water and feed at the lower 2 levels, and a nestbox at the upper level (Figure 2). Enclosure doors on the lower tier opened each morning allowing hens to access litter-covered floor areas in front of and underneath the tiered enclosure (Figure 2). Within each enclosure unit were 8 round metal perches (3.18 cm diameter; Figure 2), each extending the full length of the unit (235 cm). Three perches were present in the lower level, 4 in the middle level, and the final perch was located on the upper level in front of the nestbox. At the stocking rate used in this study hens were provided the required 15 cm/hen of perch space (United Egg Producers, 2010) and all with a minimum 20 cm distance between perch and ceiling (Struelens and Tuyttens, 2009; Figure 2). Two solid metal ledges, provided to help hens transition between levels in the enclosure, ran the full length of the unit in front of the upper and middle levels (Figure 2). Each ledge was 30.50 cm wide, providing a total surface area of 7,167.50 cm^2^ within the unit enclosure (e.g., 50.48 cm^2^ for each of the 142 hens with which the unit was initially stocked). Adjacent to the upper tier perch, the nestbox, which also ran the length of the unit, was 52.16 cm wide providing 12,257 cm^2^ of nesting space per unit (e.g., 86.32 cm^2^ for each of the 142 hens with which the unit was initially stocked). The mesh floor of the upper tier extended 36.83 cm from the nestbox for a total area of 8,655.05 cm^2^ within the enclosure unit; however, some of this area was located under the upper tier perch.

### Data Collection

Live observations of hens’ use of perches and ledges inside the tiered enclosures were conducted over 2 consecutive days at each of 3 age points in the laying hen production cycle: peak lay (24 wk; 96.5% production), mid lay (55 wk; 89.33% production) and end lay (76 wk; 76.85% production). Cumulative mortality as a percentage of hens originally placed was recorded for the entire aviary barn (peak lay: 0.97%; mid lay: 6.03%; end lay: 10.99%), but these mortalities were not used to adjust any of our data as mortality was not reported on a per unit or section basis. Thus, all estimates of perch occupancy and crowding were based on bird numbers at population.

Live observations were conducted by 2 teams of 2 observers walking on either side of an enclosure row to simultaneously count hens on all perches and ledges within the 3 tiers of the designated enclosure units. Observers trained together by observing one unit enclosure prior to data collection and row observations were balanced between observer pairs across the 2 sets. Observations were made just prior to lights on (‘dark’: 2 sets/day), and 3 hours after lights on but before the aviary doors opened to allow hens litter access (‘light’: 2 sets/day). All 4 rows of enclosures were sampled during each observation period (both single enclosure rows and half of the 2 double rows), with 4 sections in each row observed (Figure 1). Three of the 5 to 6 possible units within each section were observed (the same units were observed across the 2 days) with selected units balanced between sections. In total, each of the 48 selected enclosure units were observed 4 times during the ‘dark’ period and 4 times during the ‘light’ period at each of the 3 hen age points. Disturbance of hens during ‘dark’ observation sets was minimized by use of green headlamps and disturbance of hens during ‘light’ observation sets was minimized by limiting barn access to include observers only. No farm personnel were typically present during the lights off period.

### Ethics

All research was approved by the Michigan State University Institutional Animal Care and Use Committee prior to the start of data collection.

### Statistical Analyses

All observations were conducted at the level of the unit, and the aviary unit was our experimental unit for statistical analyses. As described above, all observations of perch and ledge use in the enclosures were conducted when hens were confined to the enclosure and did not have litter access. As hens had the potential to redistribute themselves unevenly between units within a section when aviary doors granted them access to litter, data are generally presented with respect to how much space is occupied using 15 cm per hen for linear perch space (United Egg Producers, 2010) and 318 cm^2^ (ledge length × width) lying down space per hen for ledges (Mench and Blatchford, 2014). To assess differences in perch use and ledge use, between ‘dark’ and ‘light’ periods, we averaged data from the 3 sampled units (average percentage of space occupied across the 3 unit tiers) within each section to create one data point for each focal section representing hens perching on a per unit basis for ‘dark’ (n = 16) and ‘light’ (n = 16) observation times, averaged across the lay cycle for perches and ledges separately. To assess differences in perch use and ledge use over the lay cycle, we averaged data from the 3 sampled units for each focal section for both ‘dark’ and ‘light’ observation times to give a single average on a per unit basis for that age point (n = 16) for perches and ledges separately. To assess patterns of specific perch and ledge use, observations from the 3 sampled units within a focal section were averaged to generate one count for each roosting location (perches and ledges) for each diurnal observation period (‘dark’ and ‘light’) at each age point (peak lay, mid lay and end lay). We also averaged perching observations (excluding ledge use as the lower tier had no ledges present) from each sampled unit within focal sections to generate one count for each enclosure tier (lower, middle and upper) for each diurnal observation time period (‘dark’ and ‘light’) at each age point (peak, middle and end lay). Comparisons were made using one-way or repeated measures ANOVAs with Student's *t*-tests applied to the least squares means. All analyses were conducted in JMP 11.1.1 (SAS Institute Inc., Cary, NC) with α set at 0.05. Unless otherwise indicated, data are presented as LSM ± SEM.

Box plots were generated by JMP, and unless otherwise stated, lines within the boxes represent the median while the lower and upper boundaries of the box represent the interquartile range (i.e., difference between the first and third quartiles). The whiskers extend from the boxes to the outermost data point that falls within distances computed as follows: upper whisker = 3rd quartile + 1.5 × (interquartile range) and lower whisker = 1st quartile - 1.5 × (interquartile range). If data points do not reach the computed ranges, then the whiskers are determined by the upper and lower data point values (not including outliers). The disconnected points are potential outliers.

## RESULTS

**Figure 3.**
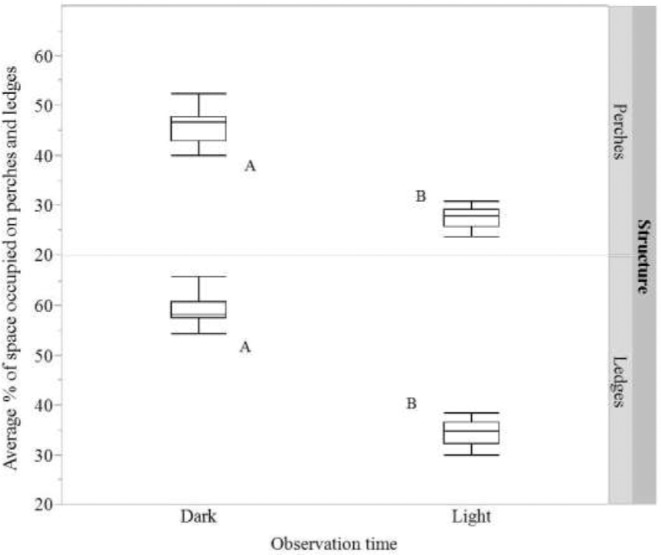
The average percentage of space occupied by hens on perches and ledges per unit for the 2 diurnal observation time periods (‘dark’ and ‘light’). Dissimilar letters indicate significant differences in space occupied by hens between time periods.

On average, a higher percentage of space on perches was occupied by hens during the ‘dark’ period than during the ‘light’ period (F_1,30_ = 277.29, *P* < 0.0001; 45.06% ± 0.83 vs. 25.54% ± 0.83, for ‘dark’ and ‘light’ respectively; hen counts per aviary unit: 54.08 ± 0.99 vs. 30.65 ± 0.99, for ‘dark’ and ‘light’ respectively; Figure 3). Similarly, on average, a higher percentage of space on ledges was occupied by hens during the ‘dark’ period than during the ‘light’ period (F_1,30_ = 706.72, *P* < 0.0001; 59.05% ± 0.65, 34.56% ± 0.65, for ‘dark’ and ‘light’ respectively; average hen counts per aviary unit: 21.98 ± 0.66, 15.47 ± 0.66, for ‘dark’ and ‘light’ respectively; Figure 3).

**Figure 4.**
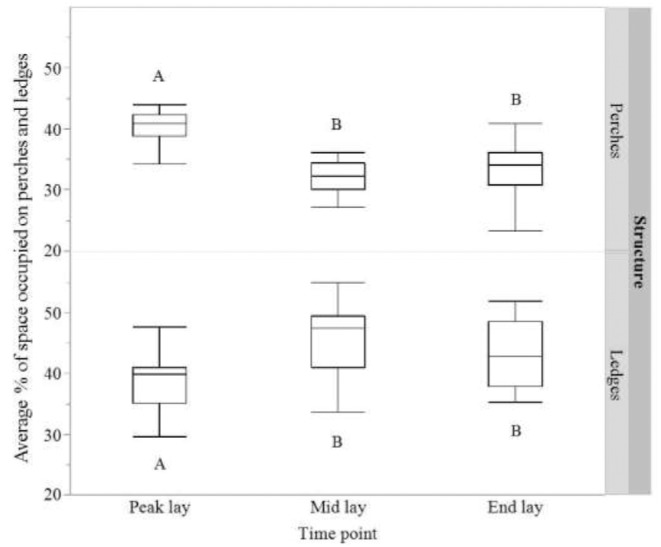
The average percentage of space occupied by hens on perches and ledges per unit at each of the 3 observed age points (peak lay, mid lay and end lay) throughout the study. Dissimilar letters indicate significant differences in space occupied by hens between age points.

A higher percentage of space was occupied on perches at peak lay than at any other age point (F_2,45_ = 30.41, *P* < 0.0001; 40.48% ± 0.82 (peak) vs. 32.11% ± 0.82 (mid) vs. 33.31% ± 0.82 (end); average hen counts per aviary unit: 48.58 ± 2.73 (peak) vs. 38.53 ± 2.73 (mid) vs. 39.98 ± 2.73 (end); Figure 4). Conversely, the lowest percentage of space was occupied on ledges at peak lay compared to mid or end lay (F_2,45_ = 7.29, *P* < 0.0018; 38.44% ± 1.44 (peak), 46.18% ± 1.44 (mid), 43.06% ± 1.44 (end); average hen counts per aviary unit: 16.91 ± 0.97 (peak) vs. 20.32 ± 0.97 (mid) vs. 18.94 ± 0.97 (end); Figure 4). No differences were observed in the percentage of perch space occupied by hens in single or double rows (F_1,14_ = 0.003, *P* = 0.95; 35.35% ± 1.02 vs. 35.26% ± 1.02, for single and double rows respectively; hen counts per aviary unit: 42.31 ± 2.31 vs. 42.42 ± 2.31, respectively) or ledge space occupied in single or double rows (F_1,14_ = 1.18, *P* = 0.30; 46.21% ± 0.78 vs. 47.41% ± 0.78, for single and double rows respectively; average hen counts per aviary unit: 18.13 ± 0.81 vs. 19.32 ± 0.81 respectively).

**Figure 5.**
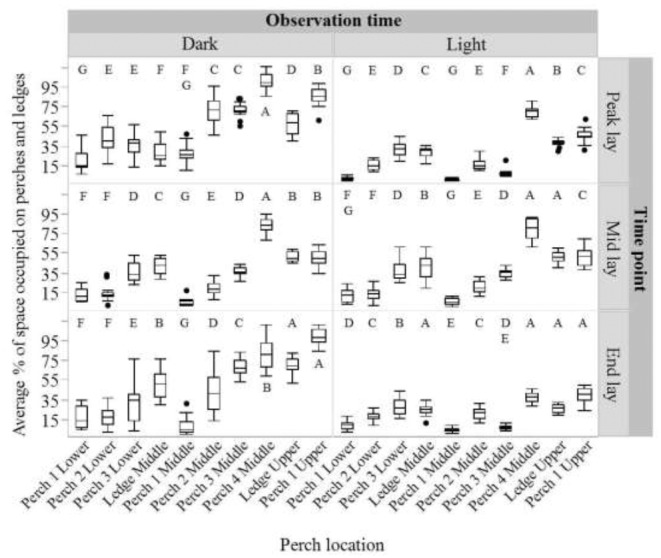
The average percentage of space occupied by hens for each perch and ledge location within the tiered aviary before lights on (‘dark’) and after lights on (‘light’) at each sampling age point (peak lay, mid lay and end lay). Dissimilar letters indicate significant differences in space occupied by hens at different locations.

**Figure 6.**
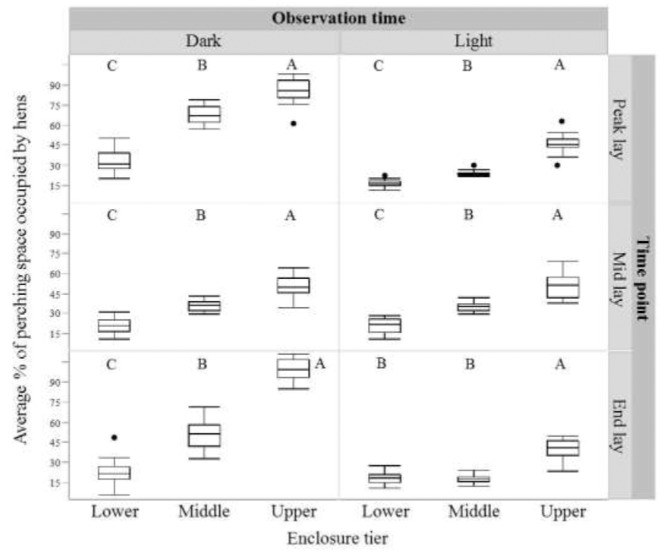
The average percentage of space occupied by hens on perches only (excluding ledges) within each enclosure tier (lower, middle, upper) before lights on (‘dark’) and after lights on (‘light’) at each sampling age point (peak lay, mid lay and end lay). Dissimilar letters indicate significant differences in the percentage of perching space occupied by hens between enclosure tiers.

There was significant variation in where hens roosted at each age point (peak, middle, end lay) and diurnal observation time (‘dark’, ‘light’; *P* < 0.001 for all age points/diurnal observation time combination). However, hens most often preferred the upper ledge, the perch in the upper tier, the middle ledge and the fourth perch of the middle tier (Figure 5). At both observation times (‘dark’ and ‘light’) within each age point, hens significantly preferred perches (excluding ledges) in the upper tier and occupied those perches in the lower tier (where no ledges are present) the least (except for equal preferences for the middle and lower tier during the ‘light’ observation period at end lay (all *P* < 0.001; Figure 6)).

Calculations from raw observational data (n = 1,158 total observations across the flock cycle for each roosting location) revealed that perches were overcrowded (i.e., >100% occupancy based on 15 cm/hen) in 19% of Perch 4 Middle tier observations, 10% of Perch 1 Top tier observations, 0% of Perch 1 Lower tier and Perch 1 Middle tier observations, and in less than 2% of cases for all remaining perches. (See Figure 1 for locations of each perch.) Ledges were overcrowded or at 100% occupancy in less than 1% of observations.

## DISCUSSION

This study of perch and ledge use by laying hens in a commercial aviary showed diurnal variation in where hens roosted as well as differences throughout the flock cycle as hens aged. Expected preferences of hens for perching in elevated levels were observed, leading to crowding on the middle and upper enclosure levels and empty space on the lower tier. In particular, the perching patterns observed during the ‘dark’ period, were consistent with previous laboratory experiments and observations at commercial facilities confirming hen preferences for elevated roosting overnight (Olsson and Keeling, 2000; Odén et al., 2002; Wichman et al., 2007; Streulens et al., 2008; Schrader and Müller, 2009; Brendler et al., 2014). The ‘dark’ period was also the time of highest perching synchrony.

This commercial system complied with the United Egg Producers guidelines (15 cm/hen, a minimum 20% of perch space ≥40 cm above the house floor, and perch diameter and materials that allow hens to wrap their toes around the perch and balance while resting; United Egg Producers, 2010). However, it did not appear that all perches were equally preferable to the hens. Observations in the present study showed hens sometimes chose to crowd (over 100% capacity) the middle and upper level perches while leaving the 3 perches on the lower level relatively empty. Furthermore, many hens roosted on the 2 elevated ledges. Based on kinematic analysis of HyLine W-36 hens of a similar body weight to hens in this study (1.5 to 1.6 kg during observation periods), each ledge was estimated to be able to hold 22 hens lying down or, more conservatively, 12 hens if the birds were standing (Mench and Blatchford, 2014). Thus, it may be beneficial to consider whether system designs can be developed that provide more perches higher in the system; perhaps by moving other resources such as nestboxes to lower levels or separating them from the aviary tiers (Lentfer et al., 2011).

The lack of preferable perching space may also become exacerbated in aviary systems, such as this one, that allow co-mingling of hens from multiple enclosure units during the day in a shared litter area within a larger aviary section (5 to 6 enclosure units per fully separated section). Hens are unlikely to evenly redistribute themselves among units when they move from the shared litter area back into tiered enclosures at night, particularly if some units are more attractive than others. Although we did not perform total counts of hens in each unit within a section, personal observations indicated some enclosure units were more crowded than others. Further research should count hens after re-entry into the system at night to help document evenness of distribution. Such work could also provide an indication of hen preferences for specific units within aviary sections, whether patterns are related to social factors (groups of hens roosting together) or spatial preferences (e.g., end units), and if preferences for certain enclosure units are consistent between sections and across time.

Perches in non-cage systems have recently been defined as structures that are elevated from the group, offer a vantage point for surveying surroundings, and that birds can grasp with their feet (EFSA AHAW Panel, 2015), with some guidelines specifying that toes be able to wrap around the structure to enable a balanced, relaxed posture for an extended period of time (United Egg Producers Guidelines, 2010; but see Schrader and Müller, 2009). By this definition, perches could include graspable edges of tiered floors or ledges; and grasping has previously been shown to improve leg bone strength (Duncan et al., 1992; Barnett et al., 1997). However, evidence comparing bone density between hens housed in furnished cages (with perches) and this aviary system, or other non-cage systems showed lower bone density in hens housed in the furnished cages (Regmi et al., 2015; Rodenburg et al., 2008). This evidence suggested that the loading activities (e.g., jumping from ledge to ledge) of hens as they moved through a complex multi-tiered system was more beneficial than perching alone with respect to bone density. Thus, hens in aviaries may not need to specifically grasp perches for bone-strengthening benefits. Furthermore, previous studies show elevated structures to be preferable to perches per se (Schrader and Müller, 2009). Alternatively, other factors such as bird health, may be causing hens to select elevated ledges (Stratmann et al., 2015b).

Perch use may affect and be affected by the health condition of the birds – specifically keel bone damage and footpad dermatitis. Keel bone damage and foot dermatitis may both be caused by perch use (Struelens and Tuyttens, 2009), or by other variables within the system (e.g., collisions with system structures or hens (Wilkins et al., 2011; Campbell et al., 2015) or wet litter (Wang et al., 1998)). These conditions may result in reduced perch use across the lay cycle as hens may try to roost in an elevated location while minimizing localized pressure on the feet and keel (Struelens and Tuyttens, 2009; Pickel et al., 2011). Welfare Quality^®^ scoring on a sample of hens in this commercial system showed an increase in both severity of foot lesions and incidence of keel damage across the flock cycle (Blatchford et al., 2015). This may account for the high use of the middle tier ledge (solid flat metal), and the increasing use of ledges and decreased use of perches across the lay cycle. Finally, personal observations indicated many hens perched on the edge of the wire tier floor in the upper tier directly in front of the perch. This appeared to occur even when the upper perch was not fully occupied, although we did not count these hens in this study, as only use of true perches was formally evaluated. Further research should identify all locations hens use for roosting within the aviary enclosures, particularly at night during highest perching synchrony, and how roosting preference changes across the lay cycle may be correlated with changing bird health.

Previous studies have indicated hens will show unrest without perch access (Olsson and Keeling 2000) and are motivated to work to gain perch access (Olsson and Keeling 2002), however, perches can also have detrimental effects on health parameters (Struelens and Tuyttens, 2009; Hester et al., 2013;) and thus, their presence may actually impair welfare. Furthermore, crowding on some perches may increase frequencies of falls or collisions between hens, which could cause injuries such as keel fractures. The incidence and severity of perch-related health issues may be modified by perch design including perch softness (Pickel et al., 2011; Stratmann et al., 2015a) and perch shape (Struelens and Tuyttens, 2009) with lower perches made of a more appealing material (e.g., textured variable wood simulating tree branches) to encourage higher use of them. Further tests of commercial applications in different non-cage system designs are warranted, including documenting effects across the lay cycle of several flocks within different strains of birds and subsequent effects on individual hen health and welfare.

Overall, this research in a commercial aviary system reinforces the claim that simply providing sufficient total perch space may not be enough to improve welfare. Perches must be provided in a way that allows all hens’ equal access to preferred perches, particularly at night when hens are most motivated to use them. Furthermore, hens may prefer to use alternative structures in the system, such as ledges or the mesh edges of the uppermost tier for roosting, which may be influenced by the health of the hens across the lay cycle.
